# Whole-genome sequencing of Staphylococcus capitis isolates from neonates with healthcare-associated bacteraemia in neonatal intensive care units in Taiwan: comparison between the NRCS-A strain and a non-NRCS-A multi-resistant strain

**DOI:** 10.1099/mgen.0.001798

**Published:** 2026-07-29

**Authors:** Yu-Rou Yeh, Yi-Hsuan Huang, Chih-Jung Chen, Rey-In Lien, Ming-Chou Chiang, Yhu-Chering Huang

**Affiliations:** 1 Department of Medical Education, Chang Gung Memorial Hospital at Linkou, Kweishan, Taoyuan, Taiwan, ROC; 2 Department of Pediatrics, Chang Gung Memorial Hospital at Linkou, Kweishan, Taoyuan, Taiwan, ROC; 3 Chang Gung University School of Medicine, Kweishan, Taoyuan, Taiwan, ROC

**Keywords:** neonates, NRCS-A strain, pulsed-field electrophoresis, *Staphylococcus capitis*, whole genome sequencing

## Abstract

*Staphylococcus capitis* is a common pathogen causing late-onset sepsis in neonatal intensive care units (NICUs). A specific clone, *S. capitis* NRCS-A, which was first reported in 2012 from France, is spreading globally in NICU settings. The present study was carried out to explore the molecular characteristics and clinical features of *S. capitis* bacteraemia among infants hospitalized in NICUs in Taiwan. We retrieved and molecularly characterized 191 *S. capitis* bloodstream isolates from hospitalized patients of a medical centre in Taiwan between 2014 and 2021, with whole-genome sequencing (WGS) for 79 isolates. Seventy-three isolates were identified from 65 infants in neonatal units and 118 isolates from inpatients staying outside the neonatal units (non-neonates). Seventy-one (97%) of the 73 neonate isolates were characterized to 2 major pulsotypes: 16 out of 73 (22%) were pulsotype 300 which was similar to that of the NRCS-A strain, and 55 isolates were pulsotype 1300, while 27 pulsotypes were identified from 118 non-neonatal isolates. Seventy (96%) of 73 neonatal isolates were oxacillin-resistant. Of the 73 neonatal isolates and 6 selective non-neonatal isolates (pulsotype 300 for 5 isolates) selected for WGS, 69 neonatal isolates and 5 non-neonatal isolates clustered into four major clades (A to D), corresponding to the two major pulsotypes, 300 and 1300. By phylogenetic analysis, 15 neonatal isolates and 5 non-neonatal isolates, sharing pulsotype 300 and clades C/D, were embedded within the international isolates of the previously designated pandemic outbreak clone of the NRCS-A strain and also carried NRCS-A strain-related marker genes, confirmed as the NRCS-A strain. In addition to resistance to oxacillin and penicillin, the dominant clone (non-NRCS-A strain) exhibited additional resistance to erythromycin and clindamycin. Conclusively, in contrast to diverse genetic characteristics among non-neonatal isolates, over 95% of *S. capitis* bloodstream isolates from neonates in Taiwanese NICUs were resistant to oxacillin and belonged to two major clones, including the NRCS-A strain. The dominant clone was a non-NRCS-A strain and exhibited broader antibiotic resistance. Further surveillance is needed.

**Impact Statement**

*Staphylococcus capitis* is among the common coagulase-negative staphylococci causing late-onset sepsis in neonatal intensive care units (NICUs). In 2012, a specific clone of *S. capitis*, designated as NRCS-A, was first identified and reported from NICU patients in France as the same pulsotype. This strain was subsequently identified in numerous NICU settings globally. In Taiwan, though an iconic study published in 1999 first pointed out that a high proportion of *S. capitis* isolates were multidrug resistant, no further studies were published on this issue subsequently. In this work, we retrieved a total of 191 isolates of *S. capitis* bloodstream isolates from inpatients between 2014 and 2021. In contrast to diverse genetic characteristics (27 pulsotypes) among 118 non-neonatal isolates, 71 (97%) of the 73 isolates from neonates were characterized to two major pulsotypes. Whole-genome sequencing revealed that 69 of 73 neonatal isolates were clustered into 4 major clades, corresponding to the 2 major pulsotypes, and 15 (20.5%) neonatal isolates sharing the second major pulsotype were embedded within the international isolates of previously designated pandemic outbreak clone of NRCS-A strain and also carried NRCS-A strain-related marker genes, confirmed as NRCS-A strain. A non-NRCS-A strain, which carried type IIIa SCC*mec* and harboured more significant numbers of resistance genes (e.g. *erm*A, *dfr*C, *fus*B, *mec*I and *mec*R1), was predominant and exhibited broader antibiotic resistance. Further surveillance is needed.

## Data Summary

The assembled draft genomes and raw paired-end FASTQ sequencing reads of all 79 local *S. capitis* are deposited at under NCBI BioProject PRJNA1250610. Raw reads were submitted to NCBI SRA (BioSample accession numbers: SAMN47947204–SAMN47947282).

## Introduction

Coagulase-negative staphylococci (CoNS) were once considered apathogenic bacteria of healthy human skin and mucosa [1]. However, CoNS have emerged as a leading cause of healthcare-associated infections in hospitalized patients, especially among preterm infants staying in neonatal intensive care unit (NICU) who often require more invasive medical care [1–4]. While *Staphylococcus epidermidis* has long been regarded as the major late-onset sepsis (LOS)-associated CoNS in NICU, *Staphylococcus capitis* has been also reported in several studies among the frequently isolated micro-organisms [4–16]. *S. capitis* was reported by some investigators to be associated with more severe morbidity than non-*S. capitis*, CoNS [5, 13], but this scenario was not observed by others [17].

In 2012, a specific clone of *S. capitis*, designated as NRCS-A, was first reported from NICU patients in France as a same PFGE pattern [13]. NRCS-A isolates have a multidrug resistance profile, including methicillin, aminoglycosides and fosfomycin, and some isolates are heteroresistant to vancomycin. Most NRCS-A isolates carry an SCC*mec* mobile genetic element which harbours *mec*A, responsible for the methicillin resistance, and usually type V SCC*mec* [13]. This strain was subsequently found in 154 isolates identified between 1994 and 2015 from 34 NICUs in 17 countries [15, 16]. Since identified, the NRCS-A strain has evolved into 3 lineages (‘proto-outbreak 1’, ‘proto-outbreak 2’ and ‘international outbreak’), and 59 genes specific to the NRCS-A clone were identified as markers through genomic comparisons to allow sequence-based detection of this clone [5, 16].

In Taiwan, CoNS are a leading cause of LOS in NICUs, accounting for ~40% of LOS aetiologies in several large-scale studies [18–20]. However, most studies did not identify CoNS isolates to the species level. An iconic study conducted in a Taiwanese NICU [6], which collected 236 positive blood cultures from 1995 to 1997, first pointed out that a high proportion of *S. capitis* isolates were multidrug resistant (MDR). No further studies were published on this issue subsequently. With the introduction of MALDI-TOF MS in our hospital in 2013, most clinical CoNS isolates can now be identified to the species level, facilitating the possibility of molecular epidemiological studies on this pathogen. Given the limited studies on the molecular epidemiology of *S. capitis* in neonates in Taiwan, as well as the correlations between clinical features and molecular characteristics of *S. capitis* bacteraemia in neonates, we conducted this study. We molecularly characterized, including through whole-genome sequencing (WGS), *S. capitis* bloodstream isolates from infants hospitalized in our NICUs and then compared clinical features and antibiotic susceptibility among genetically stratified isolates. We also conducted a phylogenetic analysis and comparison between 250 *S. capitis* global strains from the Wirth *et al*. [16] study and local *S. capitis* isolates from Taiwan. The 250 global *S. capitis* strains from Wirth *et al*. [16] were selected as the comparator dataset because they represent the largest publicly available collection of *S. capitis* genomes specifically from NICU settings, include isolates with established NRCS-A and non-NRCS-A lineage designations and have been used as the standard reference comparator in subsequent *S. capitis* genomics studies [14, 21].

## Methods

### Subjects and setting

Using the microbiologic database of Chang Gung Memorial Hospital (CGMH) at Linkou, we retrieved *S. capitis* bloodstream isolates from all hospitalized patients, including neonates and inpatients staying outside the neonatal units (non-neonates, from children to elderly), between January 2014 and December 2021. These isolates were systematically collected, preserved and stored at our hospital. An infant with one or more bloodstream *S. capitis* isolates, which were identified from blood cultures obtained at least 72 h after admission to NICUs and were also available for further analysis, was enrolled in this study.

This study was conducted at Chang Gung Paediatric Medical Center, a part of CGMH at Linkou, a university-affiliated teaching hospital in northern Taiwan, which has a total of more than 3,000 beds. The centre has five neonatal units, including three NICUs and two special care nurseries (SCNs), distributed across two floors. NICU-1 (3L) has 16 beds, NICU-2 (5L) has 24 beds, and NICU-3 (3L) has 10 beds. SCN-1 (3L) has 24 beds, and SCN-2 (5L) has 30 beds.

The study was approved by the institutional review board of CGMH (reference No: 202102234B0), and a written informed consent was waived due to the nature of the electronic medical records reviewed retrospectively.

### Microbiologic methods

In our NICUs, blood cultures were drawn from patients with suspected sepsis at the discretion of attending physicians or residents on duty. Blood specimens were collected after aseptic preparation and cultured in trypticase soy broth (Becton Dickinson, MD, USA). Single colonies grown on agar plates were selected for further analysis. *S. capitis* was identified based on colony morphology, coagulase testing and MALDI-TOF MS (Bruker Daltonics GmbH, Bremen, Germany).

### Antibiotic susceptibility study

The antimicrobial susceptibility of all *S. capitis* isolates to nine antibiotics, including clindamycin, erythromycin, oxacillin, penicillin, trimethoprim/sulfamethoxazole (TMP-SMX), teicoplanin, vancomycin, linezolid and rifampin, was tested using the disc diffusion method in accordance with the guidelines of the Clinical and Laboratory Standards Institute [22].

### Molecular characterizations

All of the *S. capitis* isolates were characterized by PFGE, staphylococcal chromosome cassette *mec* (SCC*mec*) type and the detection of the Panton–Valentine leukocidin (PVL) gene. Though the discriminatory power of PFGE is lower than that of WGS, the expense of PFGE is lower than that of WGS here in Taiwan. In addition, the specific clone of *S. capitis*, namely NRCS-A, was first identified as a same PFGE pattern and designated as such, so we first performed PFGE to molecularly characterize all the collected *S. capitis* isolates in this study and then selectively for further WGS.

PFGE with *Sma*I digestion was performed according to the procedure described previously [23, 24] and analysed by both the manual method according to the criteria proposed by Tenover *et al*. [25] and digitalized method. PFGE patterns with four or more band differences were classified as different types. The pulsotype was designated in Arabic numbers; any new type, if identified, was designated consecutively. PFGE patterns with less than four-band differences from an existing genotype were defined as subtypes. The BioNumerics software program (Applied Maths Scientific Software Development, Sint-Martens-Latem, Belgium) was also applied for cluster analysis using the Dice coefficient and the unweighted pair-group method.

Staphylococcal chromosomal cassette mec (SCC*mec*) typing was determined by multiplex PCR strategies described previously [26]. The presence of PVL genes was determined by a conventional PCR strategy described previously [27].

### WGS analysis

After cultured on blood agar incubated overnight, colonies of each test *S. capitis* isolate were lysed and digested, and then, the chromosomal DNA was extracted using Tissue and Cell Genomic DNA Purification Kit (GeneMark, TAIWAN). We used a MiSeq sequencer from Illumina (San Diego, CA, USA) to perform WGS. Sequencing libraries were prepared using the Nextera XT DNA Library Prep Kit (Illumina) and sequenced with 2×301 bp paired-end reads. Sequence data were analysed using Bactopia v3.0.0 [28] run via Nextflow v23.04.3. Within the Bactopia pipeline, raw reads were quality-filtered using fastp v0.23.4 with automatic paired-end adapter detection (default settings: minimum read length 15 bp, qualified base quality ≥Q15, reads discarded if >40% of bases fall below Q15). PhiX contaminants were subsequently removed using BBDuk v39.01 (ref=phix, k=31, hdist=1, qtrim=rl, trimq=6, minlength=35, minavgquality=10). Reads were then subsampled to a target of 250 Mb per isolate (~100× genome coverage) using BBMap ReformatReads v39.01. All 79 isolates passed QC; mean post-QC sequencing depth was 300× ± 36× (range 237–451×) relative to the CR01 reference genome (2,522,871 bp). For *de novo* assembly, the Shovill pipeline incorporated in the Bactopia pipeline was utilized. It employed SKESA v2.4.0 as its core assembler, with assemblies polished using Pilon v1.24. The resulting contig counts varied from 9 to 39 across all 79 assembled genomes, with N50 and L50 values ranging from 156,724 to 1,291,113 and from 1 to 5, respectively. The following software versions were used: fastp v0.23.4, BBDuk/BBMap v39.01, Shovill v1.1.0 with SKESA v2.4.0 and Pilon v1.24, Prokka v1.14.6, Parsnp v1.1 [29], Gubbins v3.3.1 [23] with RAxML v8.2.12 (GTRGAMMA model, 5 iterations), ARIBA v2.14.6 with CARD (Comprehensive Antibiotic Resistance Database) v3.1.0 (maximum ARO accession 3004836, accessed via Bactopia v3.0.0) and VFDB full set (accessed March 2024), PlasmidFinder v2.1.6 with Gram-positive replicon database (accessed March 2024), staphopia-sccmec v1.0.0, AMRFinderPlus v3.11.18 (database v2023-08-08.2), AGRvate v1.0.2 and iTOL (itol.embl.de) for tree visualization. Both the assembled draft genomes and raw paired-end FASTQ reads for all 79 isolates are deposited under NCBI BioProject PRJNA1250610 (see Data Summary).

Multi-alignment of the core genomes was conducted with the Parsnp procedure, and the multi-alignment file was subsequently used for maximum likelihood phylogeny construction based on the SNPs outside of the potential recombination regions with the Gubbins procedure [29, 30]. CR01 (GCF_000499705.1) was used as the reference strain, and the multi-alignment file’s final length was 2,160,756 nucleotides. After creating a final phylogenetic tree, we input the data into Interactive Tree of Life (http://itol.embl.de) for further annotation. To better understand the genetic relationship between strains from Taiwan and those from other parts of the world, the raw sequencing reads for the 250 global *S. capitis* strains from the Wirth *et al*. [16] study were downloaded from NCBI SRA (BioProject PRJNA493527) and processed through the same Bactopia v3.0.0 pipeline used for the local strains, including identical QC, *de novo* assembly (Shovill/SKESA v2.4.0/Pilon v1.24) and annotation (Prokka v1.14.6) steps. All 329 resulting assemblies (250 global+79 local) were included simultaneously in the Parsnp core-genome alignment using CR01 (GCF_000499705.1) as the reference genome.

### Detection of plasmids, resistance and virulence genes and SCC*mec* types

The Bactopia tools were utilized to detect the harboured plasmids and genes encoding resistance and virulence. The ariba module, along with CARD and VFDB (http://www.mgc.ac.cn/VFs), was employed to identify the resistance and virulence genes, respectively. The plasmids were screened using the PlasmidFinder module. The identification of SCC*mec* types was conducted on the assembled draft genomes with the staphopiasccmec module provided as part of the Bactopia pipeline. However, this module failed to identify type V SCC*mec*, which was identified by other methods described previously [23, 24].

### Identification of the NRCS-A strain

To identify the NRCS-A strain, we first examined and compared PFGE patterns of the studied isolates with those of the NRCS-A strain shown in previous publications [10, 11]. For those with the same or similar PFGE pattern of the NRCS-A strain, we performed further characterizations. Isolates were classified as belonging to the NRCS-A lineage based on three complementary criteria: (1) phylogenetic clustering within the NRCS-A clade in the global *S. capitis* phylogeny of Wirth *et al*. [16]; (2) carriage of NRCS-A-specific marker genes — to screen for these, the 59 marker gene sequences defined by Wirth *et al*. [16] were retrieved from the original CR01 reference genome [EMBL accessions LN866849 (chromosome) and LN866850 (plasmid)] using Prokka annotation and screened against all the local assemblies by blastn (≥80% nucleotide identity, ≥70% query coverage); and (3) confirmation of SCC*mec* type V by multiplex PCR as previously described [23, 24].

### Data collection for clinical analysis

We retrospectively reviewed the medical records of enrolled patients and collected data on demographics, clinical manifestations, laboratory results, presence of a central venous catheter, usage of mechanical ventilation, treatment courses for bacteraemia and clinical outcomes. All data describing episodes of LOS caused by *S. capitis* were reviewed for face validity by two investigators (Y.-R.Y. and Y.-H.H.).

### Definitions

The diagnosis of *S. capitis* bacteraemia was consistent with that for *S. epidermidis* bacteraemia published elsewhere previously [31], requiring clinical signs and symptoms of sepsis and intravenous antibacterial therapy for at least 5 days after obtaining blood cultures or until death. Clinical signs and symptoms of sepsis, including fever or hypothermia, hyper- or hypoglycaemia, apnea or tachypnoea, bradycardia or cyanosis, frequent desaturation with increased requirement of ventilator support, feeding intolerance, jaundice, abdominal distension, decreased activity, skin mottling and hypotension, and occurring during the episode, were recorded.

Empirical antibiotics for both Gram-positive and Gram-negative organisms were usually prescribed at the onset of clinical sepsis. The modification of antimicrobial regimens was decided by attending physicians in charge according to the results and antibiotic susceptibility patterns of blood cultures.

Separate episodes in an individual patient were considered when *S. capitis* was identified with an interval greater than 14 days, and a 7–14-day course of appropriate antimicrobial agents was administered, with at least one negative blood culture. Persistent bacteraemia was defined as at least one consecutive positive blood culture for genetically identical *S. capitis* isolates, at least 48 h apart, after *in vitro* susceptible antibiotic therapy for at least 3 days during a single bacteraemic episode [32]. Recovery was defined as at least one negative blood culture after a 7–14-day course of *in vitro* susceptible antibiotic therapy.

All patients were followed until death or discharge. Definitions of underlying diseases and comorbidities of prematurity, including bronchopulmonary dysplasia and necrotizing enterocolitis, were based on the latest diagnostic criteria in the standard textbook of neonatology [33].

### Statistics

To analyse and compare the demographic and clinical characteristics of LOS due to *S. capitis*, patients were stratified into subgroups based on their pulsotypes if any major clones were identified. Pearson’s chi-squared test was used to compare categorical variables between groups, and an independent sample t-test was used to compare means of numerical variables between groups. A *P*-value of <0.05 was considered significant. All statistical analyses were performed using IBM SPSS version 19.0.

## Results

### Identification, antibiogram and molecular characteristics of *S. capitis* bloodstream isolates

Between 2014 and 2021, a total of 191 isolates of *S. capitis* bloodstream isolates were identified, 73 isolates from infants in neonatal units and 118 isolates from non-neonates. A total of 163 (85%) of the 191 isolates were oxacillin-resistant; of the 73 isolates from neonates, 70 (96%) were oxacillin-resistant. Distribution of yearly isolates from neonates and non-neonates is shown in Table S1, available in the online Supplementary Material. All of the 191 *S*. *capitis* isolates were susceptible to vancomycin. Compared with non-neonatal isolates, neonatal isolates had a significantly lower susceptibility to oxacillin (4.1% vs. 21.2%), clindamycin (21.9% vs. 40.7%) and erythromycin (19.2% vs. 41.5%), while a significantly higher susceptibility to rifampin (100% vs. 78%). A comparison of antibiotic susceptibility rate of 191 *S. capitis* isolates stratified by neonates or not is shown in Table S2.

**Fig. 1.**
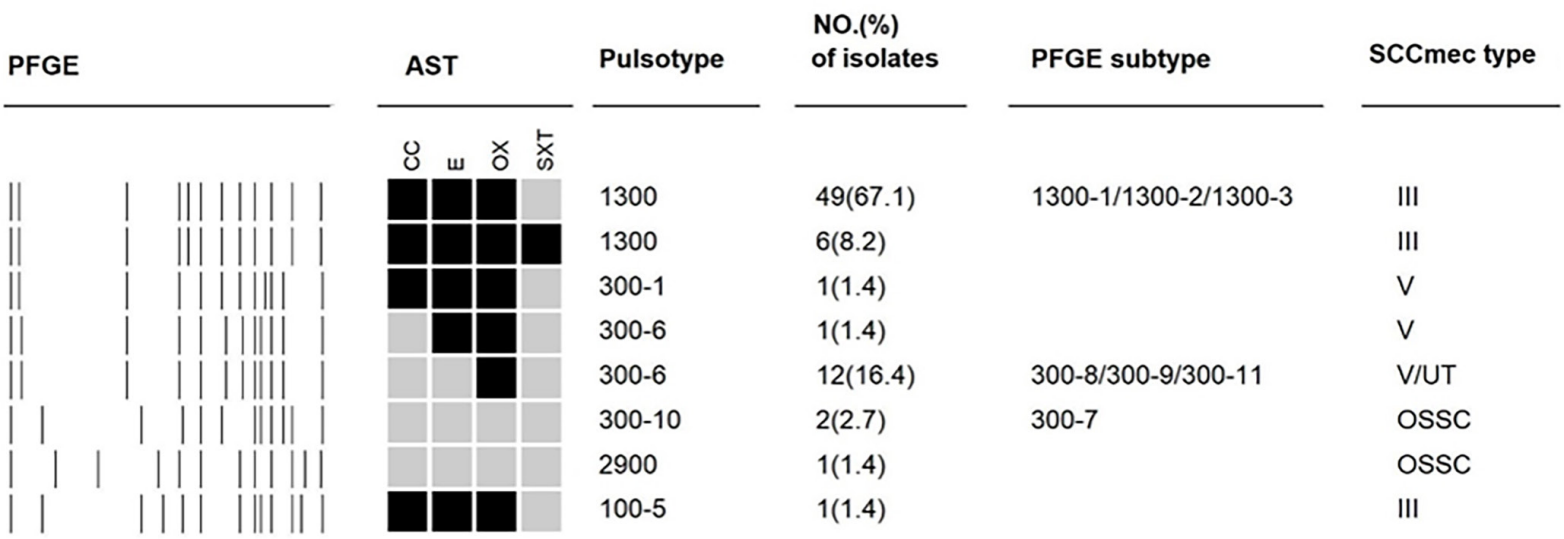
PFGE-based dendrogram, resistance profile and staphylococcal cassette chromosome (SCC*mec*) type of the 73 *S. capitis* isolates. All 73 isolates were susceptible to vancomycin, teicoplanin, linezolid and rifampin, while resistant to penicillin. None of the isolates possessed PVL genes. Antimicrobial resistance phenotypes: black indicates resistance, and grey indicates susceptibility. Abbreviations for antimicrobial resistance phenotypes are as follows: clindamycin (CC), erythromycin (E), oxacillin (OX) and TMP-SMX (SXT).

Of the 191 isolates, 3 SCC*mec* types were identified, namely types III, IV and V. None of the isolates possessed PVL genes. Of the 73 isolates from infants in NICU, 4 pulsotypes were identified by PFGE (Table 1, Fig. 1), with 2 major types, namely pulsotype 1300 accounting for 55 isolates (75.3%) and pulsotype 300 (sharing a PFGE pattern similar to the NRCS-A strain) accounting for 16 isolates (21.9%). Two other pulsotypes were singletons. All 55 isolates with pulsotype 1300 carried type IIIa SCC*mec*, while among the 16 isolates with pulsotype 300, except for 2 oxacillin-susceptible strains, the other 14 isolates carried type V SCC*mec* (Fig. 1). The distribution of yearly isolates from neonates stratified by pulsotype is shown in Table S3. Pulsotype 1300 was identified in each year during the study period; pulsotype 300 was first identified in 2017 and persistently identified throughout 2021. Whereas, of the 118 isolates from non-neonates, a total of 27 pulsotypes were identified (Table 1). There were 2 pulsotypes accounting for more than 10 isolates, pulsotype 300 for 31 isolates (26.3%) and pulsotype 800 for 16 isolates (13.6%), while 13 pulsotypes were singletons.

**Table 1.** Distribution of molecular characteristics of 191 *S. capitis* blood isolates from inpatients at CGMH between 2014 and 2021 stratified by neonates and pulsotypes

Pulsotype	No. of isolates	No. of neonates (*n*=73)	No. of non-neonates (*n*=118)	SCC*mec* type
100	9 (4.7)	1 (1.4)	8 (6.8)	UT, 3
200	1 (0.5)	0	1 (0.8)	MSSC
300	47 (24.5)	16 (21.6)	31 (26.3)	5, UT, MSSC
400	6 (3.1)	0	6 (5.1)	MSSC
500	3 (1.6)	0	3 (2.5)	UT
600	3 (1.6)	0	3 (2.5)	5
700	8 (4.2)	0	8 (6.8)	3
800	16 (8.3)	0	16 (13.6)	4, 4A
900	2 (1.0)	0	2 (1.7)	UT, MSSC
1000	1 (0.5)	0	1 (0.8)	MSSC
1100	5 (2.6)	0	5 (4.2)	5, UT, MSSC
1200	1 (0.5)	0	1 (0.8)	3
1300	56 (29.2)	55 (74.3)	1 (0.8)	3
1400	1 (0.5)	0	1 (0.8)	MSSC
1500	1 (0.5)	0	1 (0.8)	5
1600	5 (2.6)	0	5 (4.2)	5, MSSC
1700	1 (0.5)	0	1 (0.8)	3
1800	1 (0.5)	0	1 (0.8)	MSSC
1900	9 (4.7)	0	9 (7.6)	UT
2000	4 (2.1)	0	4 (3.4)	5
2100	1 (0.5)	0	1 (0.8)	3
2200	3 (1.6)	0	3 (2.5)	5
2400	1 (0.5)	0	1 (0.8)	MSSC
2500	1 (0.5)	0	1 (0.8)	MSSC
2600	1 (0.5)	0	1 (0.8)	MSSC
2700	1 (0.5)	0	1 (0.8)	5
2800	2 (1.0)	0	2 (1.7)	MSSC
2900	1 (0.5)	1 (1.4)	0	MSSC
Total	191	73	118	

MSSC, methicillin-susceptible *S. capitis*; SCC*mec*, staphylococcal cassette chromosome *mec*; UT, untypeable.

### WGS analysis

**Fig. 2.**
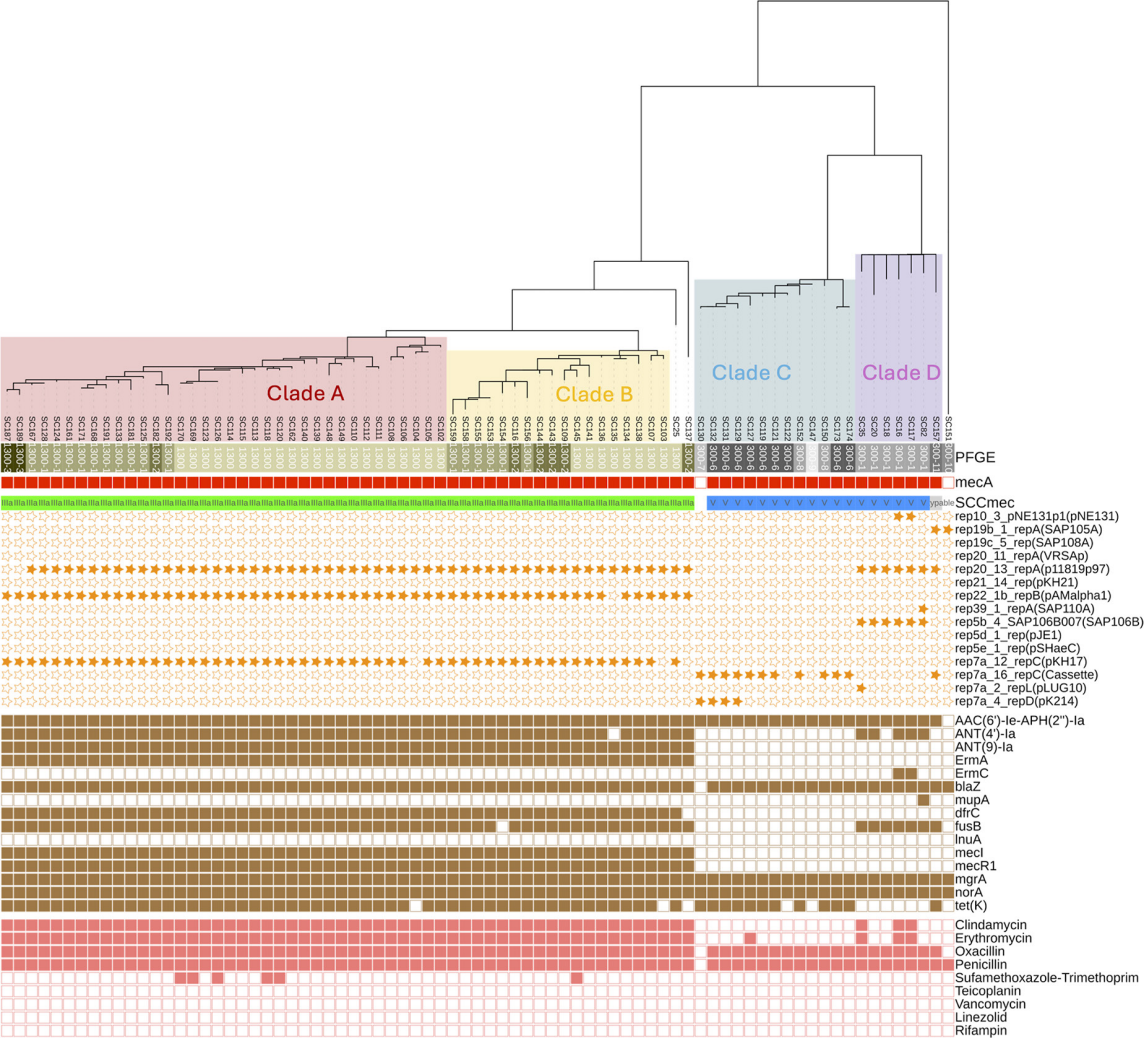
Phylogenetic analysis, plasmid carriage, resistant genes and molecular characteristics of 77 *S. capitis* strains from Taiwan. Two neonatal isolates, one with pulsotype 2900 and the other with pulsotype 100, were excluded for this analysis due to a large difference of nucleotide sequences. Four major clades of strains are identified among the strains. Each leaf node at the bottom of the dendrogram represents an individual *S. capitis* strain, identified by its unique label. Colour coding: the topmost band presents the PFGE pattern. The second band represents *mecA* gene detection by the staphopiasccmec module. The third band represents SCC*mec* types by staphopiasccmec module and a method described in ref [19, 20]. Blue colour represents SCC*mec* type V, and green colour represents type IIIa. The fourth band, the yellow-filled star, represents the presence of the plasmid by the PlasmidFinder module. The fifth band, brown square, represents the presence of the antibiotic resistance gene by fabricate module. The sixth band, pink square, represents the presence of the antibiotic resistance phenotype.

As stated in the Introduction, this study focused on neonatal population and isolates, and we thus chose a total of 79 isolates, including all 73 isolates from neonates and 6 selected isolates with major pulsotypes in neonates from non-neonates (pulsotype 300, 5 isolates; pulsotype 1300, 1 isolate), for WGS. A data list, including demographics of each patient, the year identified, antibiogram, SCC*mec* type and pulsotype of each isolate, for the 79 isolates is provided in Material S2. WGS analysis revealed that there were four major clades (A, B, C and D) identified among the 79 local isolates (Fig. 2). Four neonatal isolates, one each with one different pulsotype, and one non-neonatal isolate with pulsotype 1300, were placed outside the four major clades. Clades A (36 isolates) and B (18 isolates) corresponded to the pulsotype 1300, while clades C (13 isolates) and D (7 isolates) shared the pulsotype 300. All five pulsotype 300 isolates from non-neonates undergoing WGS belonged to clade D. The profiles of resistomes, carried plasmids and antibiograms were similar between the isolates of clade A and clade B, while the isolates of clade C and clade D harboured distinct profiles of resistomes, carried plasmids and antibiograms (Fig. 2). Within-clade genomic diversity was assessed from pairwise SNP distances computed from the Gubbins recombination-filtered core-genome SNP alignment (85,342 SNP positions, 79 local isolates). Clade A (*n*=36) showed pairwise SNP distances ranging from 0 to 55 SNPs (median 21 SNPs); clade B (*n*=18) ranged from 0 to 50 SNPs (median 27 SNPs); clade C (*n*=13) ranged from 1 to 28 SNPs (median 11 SNPs); and clade D (*n*=7) ranged from 20 to 44 SNPs (median 31 SNPs). Inter-clade distances were substantially larger, with 21–90 SNPs between clades A and B, 127–150 SNPs between clades C and D and 2,677–2,729 SNPs between clades A/B and clades C/D, confirming that the 4 clades represent genomically distinct lineages. For virulence gene detection, only genes encoding the Sdr protein, including *sdrC*, *sdrD*, *sdrE* and *sdrH*, were identified in 12 (15.2%) of the 79 strains (Table S4).

### Phylogenetic analysis and identification of the NRCS-A strain

**Fig. 3.**
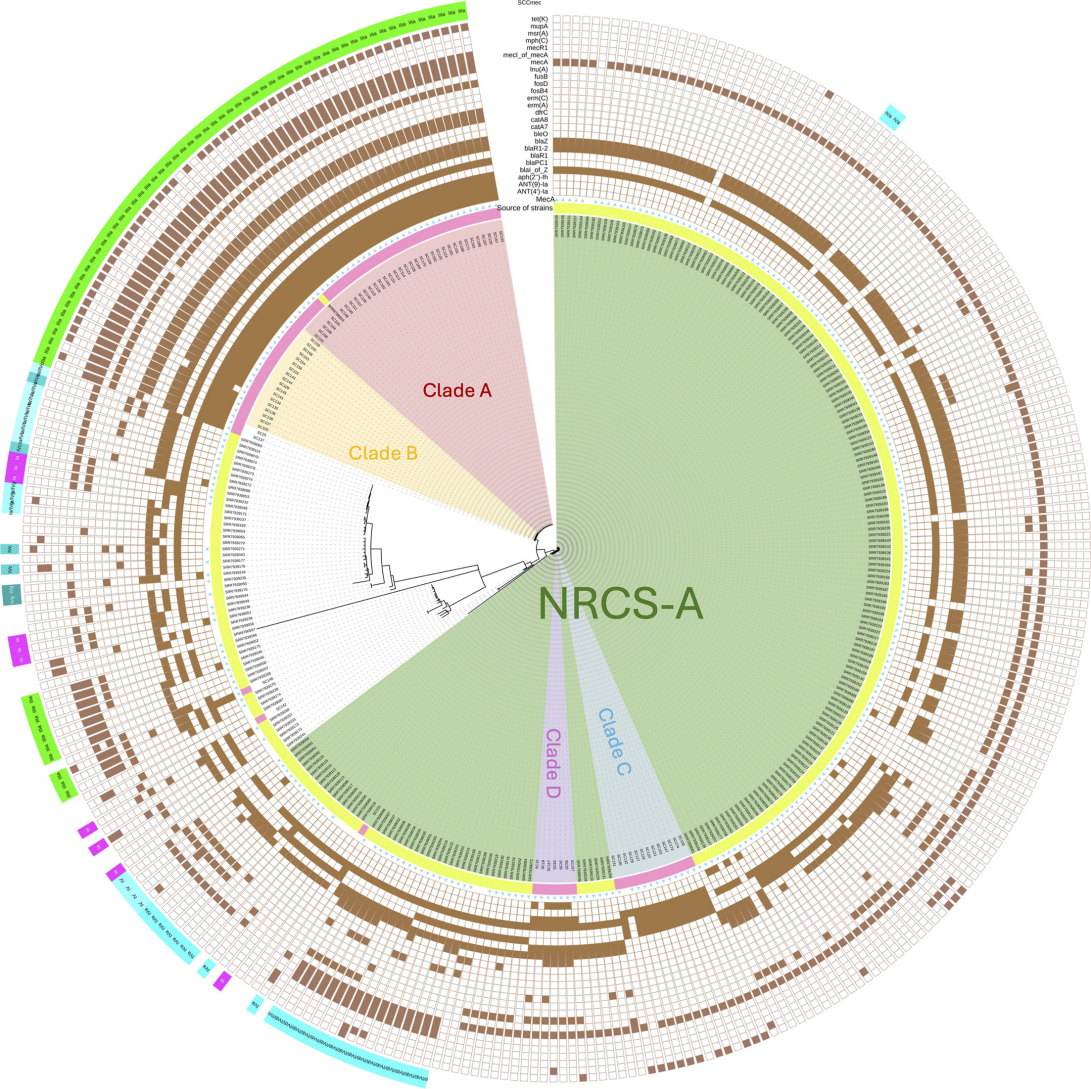
Phylogenetic analysis of 250 *S. capitis* global strains from Wirth *et al*. [16] study and 79 local strains from Taiwan. This circular dendrogram presents a phylogenetic analysis of 250 *S. capitis* strains from Wirth *et al*. [19] study and 79 local *S. capitis* strains in Taiwan based on WGS data. The dendrogram structure reveals four major clades (A, B, C and D) among the 79 local strains. Notably, local clade C and D strains are affiliated with the pandemic outbreak clone of NRCS-A. Each node on the outermost ring represents a single *S. capitis* strain. The dendrogram is colour-coded to illustrate strain origin, *mecA* gene detection, antibiotic resistance gene detection and SCC*mec* type. Colour coding: the innermost ring represents strain origin, with yellow colour representing strains from the French study and red colour representing local (Taiwan) strains. The second ring represents the *mecA* gene detection by the staphopiasccmec module, with a blue triangle representing the presence of *mecA*. The third ring represents antibiotic resistance gene detection by the fabricate module, with a brown square representing the presence of the resistance gene. The outermost ring represents SCC*mec* type by the staphopiascc*mec* module, with the green colour representing type IIIa SCC*mec*, the blue colour representing type IV or variant SCC*mec* and the red colour representing type Ia SCC*mec*, *w*hile most of the NRCS-A strains carry type V SCC*mec*, which cannot be identified by this module, and a blank colour is shown.

When the 79 local isolates were placed in a global phylogenetic context alongside 250 *S*. *capitis* genomes from Wirth *et al*. [16], local clades C and D clustered within the NRCS-A pandemic lineage (Fig. 3). To further confirm this classification, all 79 local genomes were screened for the 59 NRCS-A-specific marker genes defined by Wirth *et al*. [16] by blastn against the CR01 reference genome (EMBL LN866849–LN866850; ≥80% identity, ≥70% query coverage). Of the 59 NRCS-A marker genes, 44 could be confidently identified from the CR01 reference annotation and screened by blastn; the remaining 15 were excluded because 13 could not be confidently assigned and 2 were phage-associated markers with non-specific homologues. No clade A or clade B isolate carried any of the 44 screened marker genes (0%). In contrast, clade C isolates (*n*=13) carried the *hsdMSR* type I restriction-modification system (12/13, 92%), nisin-resistance gene *nsr* (12/13, 92%), wall teichoic acid biosynthesis genes *tar*IJL (12/13, 92%), CRISPR-Cas type III-A system (12/13, 92%) and *ccrC* recombinase (13/13, 100%); one clade C strain, SC130, was phylogenetically nested within clade C but lacked several clustered NRCS-A marker loci detected in the other clade C isolates, including CRISPR-Cas-associated and restriction-modification/nsr/wall teichoic-acid-associated regions; this pattern is consistent with possible secondary loss of these marker regions. Clade D isolates (*n*=7) carried *nsr* (7/7, 100%), *tarIJL* (7/7, 100%) and *ccrC* (6/7, 85%) but lacked *hsdMSR* (0/7) and CRISPR-Cas (0/7), consistent with a distinct NRCS-A sub-lineage. Notably, the prototype NRCS-A reference strain CR01 carries SCC*mec* IVa, whereas our clade C/D isolates carry SCC*mec* type V as confirmed by multiplex PCR – a difference previously described in Asian NRCS-A strains [12] – and accordingly, the SCC*mec* IVa-specific marker genes were not used as classification criteria. Together, phylogenetic placement, carriage of the non-SCC*mec* NRCS-A markers and SCC*mec* type V confirmation established that clades C and D, including 15 isolates from neonates and 5 isolates from non-neonates, represented the international NRCS-A lineage circulating in Taiwan.

Compared with clade C/D (NRCS-A) isolates, clade A/B isolates carried type IIIa SCC*mec* and harboured a greater number of resistance genes confirmed present by ARIBA/CARD analysis of the assembled genomes, including *ermA* (macrolide-lincosamide-streptogramin B resistance), *dfrC* (trimethoprim resistance), *mecI* and *mecR1* (beta-lactam resistance regulators) – all chromosomally located within SCC*mec* IIIa – as well as *blaZ* on the rep20-type plasmid (see Antibiotic treatment section and Discussion). Clade A/B isolates exhibited a significantly higher rate of phenotypic resistance to both clindamycin and erythromycin compared with clade C/D isolates (Fig. 2).

### Identification of LOS due to *S. capitis* in NICU

**Fig. 4.**
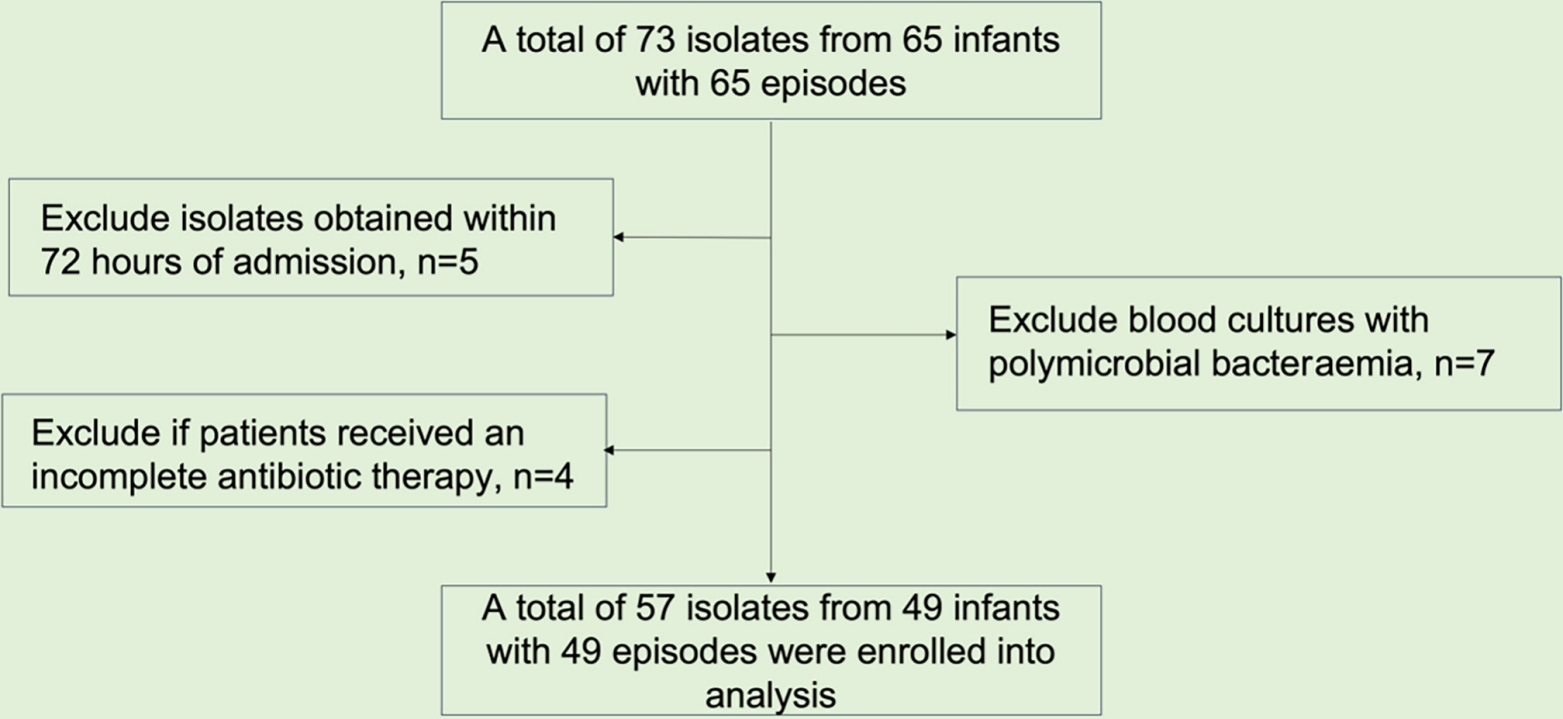
Flow chart of the isolates and patients included for the part of clinical analysis in this study.

Of the 73 *S*. *capitis* bloodstream isolates from 65 infants hospitalized in our NICUs, 16 patients (episodes) with 16 isolates were excluded for clinical analysis (details shown in Fig. 4). Of the remaining 49 patients, a total of 57 isolates from 49 patients with 49 episodes of *S. capitis* bacteraemia treated during the study period were included for further analysis.

Of the 57 isolates from 49 patients with 49 episodes, 7 episodes had multiple isolates from a single episode available for analysis. Among these, six episodes had two isolates each, and the remaining one had three isolates. Of the 6 episodes with two isolates, both isolates shared the same whole genome sequences for three episodes (Case 30, strains SC134 and SC135, isolated 8 days apart; Case 58, strains 168 and 171, isolated 2 days apart; Case 59, strains 169 and 170, isolated 2 days apart). For the other three episodes, both strains from a single case were isolated 1, 3 and 13 days apart and had one, one and five nucleotide differences, respectively. For the episode with three isolates, the first isolate (strain SC130) was oxacillin sensitive, while the other two isolates (strains SC131 and 132 identified 2 and 7 days later, respectively) were oxacillin resistant. Strain SC130 had two and one nucleotide differences from strain 131 and strain SC132, respectively. For strain SC130, the beta-lactam resistance mecA gene was absent by AMRFinderPlus, staphopia, direct BLAST, and read-depth analysis. SC130 retained only partial SCCmec IVa-associated markers, whereas SC131 and SC132 carried full-length mecA and the complete SCCmec marker set, consistent with loss or truncation/rearrangement of the mecA-containing SCCmec region rather than a SNP-induced non-functional protein.

Only one isolate from each episode was included and represented for further clinical correlation.

Of the 49 isolates from 49 patients with 49 episodes included for further analysis, only 2 pulsotypes were identified, and pulsotype 1300/SCC*mec* IIIa (non-NRCS-A strain, 85.7%) was the most common molecular characteristic, followed by pulsotype 300/SCC*mec* V (NRCS-A strain, 14.3%).

### Basic demographics

Demographic characteristics of the 49 patients with LOS caused by *S. capitis* are shown in Table 2. Males accounted for 57.1% (*n*=28/49). The mean gestational age was 28.35 weeks (range, 23–38 weeks). The average birth weight was 1,143 g (range, 500–3,020 g). The age of onset ranged from 7 to 145 days with a mean of 37 days. Peripherally inserted central venous catheters were used at the onset of bacteraemia in 43 episodes (89.6%) and were removed/replaced after the onset of bacteraemia in 31 episodes (72.1%). Twenty-three removed catheters were sent for bacterial culture, and positive cultures for *S. capitis* were found in six episodes (26.1%). However, these catheter strains were not available for analysis of genetic relatedness. No statistically significant differences were noted between the two pulsotypes in terms of demographic characteristics (birth weight, gestational age, etc.), neonatal morbidities and outcomes.

**Table 2.** Comparison of demographic characteristics of 49 episodes of LOS caused by *S. capitis* in infants who stayed in NICUs, stratified by PFGE

Characteristics	No. (%) of subjects			*P*-value
	Total (*n*=49)	PFGE 1300 (*n*=42) Non-NRCS-A strain	PFGE 300 (*n*=7) NRCS-A strain	
Male gender	28 (57.1)	22 (52.4)	6 (85.7)	0.214
Birth weight				
Mean±sd, g	1,143.84±579.14 (500–3,020)	1,096.86±497.40 (590–2,200)	1,425.71±940.46 (500–3,020)	0.398
<1,500 g	43 (87.8)	38 (90.5)	5 (71.4)	0.199
Gestational age				
Mean±sd, weeks	28.35±3.48 (23–38)	28.14±3.43 (23–36)	29.57±3.82 (26–36)	0.320
<28 weeks	23 (46.9)	21 (50.0)	2 (28.6)	0.424 0.258
>32 weeks	7 (14.3)	5 (11.9)	2 (28.6)	
NSD	19 (38.8)	16 (38.1)	3 (42.9)	1
RDS	42 (85.7)	37 (88.1)	5 (71.4)	0.258
Surgical history within 14 days	7 (14.3)	7 (16.7)	0 (0)	0.573
PICC usage prior to episode	43 (87.8)	38 (90.5)	5 (71.4)	0.199
PICC removal during episode	31 (63.3)	27 (64.3)	4 (57.1)	0.697
Outcome				
Persistent* Recovery	9 (18.4) 39 (79.6)	7 (16.7) 34 (81.0)	2 (28.6) 5 (71.4)	0.598 0.620
Mortality <30 days of onset	2 (4.1)	2 (4.8)	0	1

*Still positive for *S. capitis* from follow-up blood culture after* in vitro* susceptible antibiotic therapy for at least 48 h.

NSD, normal spontaneous delivery; PICC, percutaneously inserted central catheter; RDS, respiratory distress syndrome.

### Clinical and laboratory characteristics

Clinical symptoms and laboratory characteristics of *S. capitis* bacteraemia are shown in Table 3. The common symptoms recorded during the LOS episode included desaturation (49.0%), bradycardia (38.8%), apnea (22.4%), cyanosis (22.4%) and fever (16.3%). Leucocytosis was noted in 11 episodes (22.4%), and thrombocytopenia (platelet count <150,000 μl^−1^) was noted in 20 (40.8%) episodes. The mean C-reactive protein level at onset was 20.33 mg l^−1^ (normal, <5 mg l^−1^), and peak C-reactive protein over 40 mg l^−1^ was observed in 17 (34.7%) episodes. No statistically significant differences were found between the two pulsotypes in terms of clinical symptoms and laboratory data.

**Table 3.** Comparison of clinical symptoms and laboratory characteristics of 49 episodes of LOS caused by *S. capitis* in infants who stayed in NICUs, stratified by PFGE types

Laboratory data		No. (%) of subjects		*P*-value
	Total (*n*=49)	PFGE 1300 (*n*=42) Non-NRCS-A strain	PFGE 300 (*n*=7) NRCS-A strain	
Fever	8 (16.3)	6 (14.3)	2 (28.6)	0.320
Apnea	11 (22.4)	11 (26.2)	0 (0)	0.325
Bradycardia	19 (38.8)	18 (42.9)	1 (14.3)	0.224
Tachycardia	7 (14.3)	7 (16.7)	0 (0)	0.573
Desaturation	24 (49.0)	23 (54.8)	1 (14.3)	0.098
Poor activity	4 (8.2)	2 (4.8)	2 (28.6)	0.092
Poor feeding	1 (2.0)	1 (2.4)	0 (0)	1
Cyanosis	11 (22.4)	11 (26.2)	0 (0)	0.325
Abdominal distention	6 (12.2)	6 (14.3)	0 (0)	0.574
Tachypnea	6 (12.2)	5 (11.9)	1 (14.3)	1
WBC (×10^3^ µl^−1^) onset, mean±sd	10.09±5.54 (2.2–33.8)	10.50±5.80 (2.2–33.8)	7.67±2.83 (2.6–11.6)	0.215
Leucocytosis*	11 (22.4)	8 (19.0)	3 (42.9)	0.178
Leucopenia^†^	10 (20.4)	8 (19.0)	2 (28.6)	0.620
Hb (g/dl) Onset, mean±sd	11.53±1.72 (7.4–14.8)	11.63±1.72 (7.4–14.8)	10.96±1.73 (9.5–14.1)	0.345
Hb<10 g dl^−1^	20 (40.8)	17 (40.5)	3 (42.9)	1
Platelet (×10^3^ µl^−1^) Onset, mean±sd	243.44±140.68 (27–746)	243.05±142.58 (27–746)	245.71±139.62 (71–469)	0.964
Platelet<150×10^3^ µl^−1^	20 (40.8)	16 (38.1)	4 (57.1)	0.422
CRP (mg/l) Onset, mean±sd	20.33±30.10 (0.2–154.8)	20.35±28.22 (0.2–154.8)	20.25±43.55 (0.46–109)	0.994
CRP (mg/l) Peak, mean±sd	34.80±38.29 (0.2–154.8)	32.89±38.01 (0.2–154.8)	45.76±41.03 (0.46–100)	0.418
CRP>40 mg l^−1^	17 (34.7)	13 (31.0)	4 (57.1)	0.217
O_2_ saturation Nadir, mean±sd	77.85±16.82 (31.1–97.4)	77.30±15.55 (31.6–97.4)	81.93±25.42 (31.1–95.9)	0.433

*Leucocytosis was defined as WBC count >15,000 μl−1 in patients older than 1 month and >30,000 µl−1 in patients younger than 1 month.

†Leucopenia was defined as WBC count <5,000 µl−1.

### Antibiotic treatment

Table S5 shows a comparison of antibiotic susceptibility of the 49 neonatal *S. capitis* isolates, stratified by PFGE types (pulsotypes). All the 49 isolates were susceptible to teicoplanin, vancomycin, linezolid and rifampin and were all resistant to oxacillin and penicillin. Vancomycin minimum inhibitory concentration tests were conducted, and the results were consistent (≤ 1 µg ml) across all isolates, irrespective of pulsotypes. However, for erythromycin and clindamycin, pulsotype 1300 (clades A/B, non-NRCS-A) and pulsotype 300 (NRCS-A strain) showed considerably divergent susceptibility, 0% against 85.7% and 0% against 100%, respectively (both *P*-value=0). As for sulfamethoxazole-trimethoprim, pulsotype 1300 and pulsotype 300 showed a resistant rate of 16.7% and 0%, respectively, without significant differences. WGS-based resistance genotyping corroborated the phenotypic susceptibility results. Universal oxacillin resistance in both pulsotype groups was mediated by *mecA*, located within SCC*mec* IIIa (clades A/B) or SCC*mec* V (clades C/D). The complete resistance to erythromycin and clindamycin in pulsotype 1300 (clades A/B) isolates was attributable to *ermA*, carried within the SCC*mec* IIIa element. Penicillin resistance in clades A/B was associated with *blaZ* (penicillinase) on the rep20-type plasmid (~30–36 kb), along with its regulators *blaI/blaR1*. The *tet(K)* gene (tetracycline efflux, on the small rep7a-type plasmid, ~5 kb) was detected mainly in clades A, B and C. The *dfrC* (trimethoprim) and *ermA* resistance genes were detected exclusively in clade A/B isolates, consistent with their broader resistance phenotype.

Of the 49 episodes, empirical combined antibiotic therapy was administered in 38 episodes (77.6%), while a single empirical antibiotic (including first-generation cephalosporin, aminoglycoside or glycopeptide) was used in the remaining 11 episodes (22.4%). Inadequate empirical antibiotic therapy, defined as not including *in vitro* susceptible antibiotics, was noted in 17 episodes, with oxacillin (a 100% *in vitro* resistant antibiotic in our results) used in 10 episodes (Table S6).

Definitive antibiotic therapy with a vancomycin-containing regimen was used in 45 episodes (91.8%), and teicoplanin in 1 episode (2.0%). Three patients did not receive definitive treatment, two of them due to follow-up blood cultures revealing negative results, and one patient who passed away before susceptibility test results were available (Table S7).

Despite the administration of vancomycin for at least two consecutive days, persistent bacteraemia was detected in nine patients (18.4%). Two of these patients were infected with pulsotype 300 (clades C/D; NRCS-A strain). Eight patients used vancomycin as definitive treatment (one patient continued receiving teicoplanin treatment) for a duration ranging from 7 to 18 days. Of the nine patients, one patient did not have a follow-up blood culture within 14 days, and the rest eventually had documented negative blood culture results, ranging from 4 to 13 days. Moreover, one patient with pulsotype 1300 infection still exhibited cerebrospinal fluid positive for this strain after 3 days of teicoplanin usage, and another patient developed ascites infection after 4 days of vancomycin administration. No statistically significant differences were observed between the two groups in terms of the antibiotics used and outcomes. Overall, four infants with pulsotype 1300 infection died during hospitalization, and two of them died within 30 days of bacteraemic onset.

## Discussion

Results from this study indicated that more than 5% of *S. capitis* bloodstream isolates from infants hospitalized in neonatal units in our hospital were clustered in two clones, designated as pulsotype 1300 (major clone) and pulsotype 300 (second major clone). Whereas, a very diverse genetic background (27 pulsotypes) was identified among those from non-neonates, and nearly half of the pulsotypes were eventually singletons. This finding, clonal dissemination among neonates but not among adult patients, was similar to that reported previously from other countries, including France [9, 13, 14, 21].

Further WGS analysis and a phylogenetic analysis, including 250 global *S. capitis* isolates from the Wirth *et al*. [16] study, demonstrated that local strains of the second major clone, clades C and D (pulsotype 300), were immersed into the NRCS-A pandemic lineage (Fig. 3), implying that the international MDR-*S. capitis* strain of NRCS-A did exist in our hospital in Taiwan. However, unlike previous studies, which identified the NRCS-A strain as the dominant strain in NICU, we identified another major clone, clades A and B (pulsotype 1300), prevailing in our NICU, which accounted for nearly three quarters of the isolates from neonates, while only one isolate was identified from non-neonates. This strain, pulsotype 1300, carries SCC*mec* IIIa, and multiple antibiotic-resistant genes, and is also more resistant to multiple antibiotics than the NRCS-A strain. This strain was identified from neonates since 2014, the first year of the current study, and was evenly identified throughout 2021, the last year of the current study. This scenario that both clones, especially the major clone (pulsotype 1300), were persistently identified from the infants staying in our NICUs throughout the study period implied that this pathogen perhaps persisted and settled in the specific environment of our NICU settings. The ability to form bioﬁlm under nutrient stress and to survive desiccation [34] plus the partial ineffectiveness of incubators’ disinfection procedures [35] are probably responsible for persistence of *S. capitis*, especially NRCS-A, inside a NICU, which were proposed and reported previously.

In addition, a linezolid-resistant *S. capitis* clone emerged and disseminated in Europe recently and was reported in 2017 [36]. Later, a similar resistant clone was also identified from China [37, 38]. Though this clone was not identified from neonatal isolates from CGMH at Linkou yet, further surveillances are needed to explore this situation in Taiwan. Plasmid analysis revealed markedly different plasmid repertoires between the two dominant clonal lineages. The non-NRCS-A clade A/B (pulsotype 1300) isolates consistently harboured three plasmid types per strain on average (Fig. 2): (1) a large rep20-type plasmid (~30–36 kb; present in 65 out of 79 strains) encoding the *blaZ* penicillinase and its regulators *blaI/blaR1*; (2) a small rep22-type plasmid (~4.5–5.0 kb; 61 out of 79 strains) carrying *aadD*1 (aminoglycoside resistance) and *bleO* (bleomycin resistance); and (3) a rep7a-type plasmid (~5.0 kb; 71 out of 79 strains) carrying *tet* (K) (tetracycline efflux pump). NRCS-A clade C isolates carried predominantly only the rep7a-type plasmid (mean 1.2±0.7 replicons; two strains harboured no detectable replicon), whereas clade D isolates showed a more diverse plasmid repertoire (mean 4.6±1.1 replicons), including rep5b-type plasmids (~8.5 kb, SAP family) and, in two strains, a rep10-type plasmid (~2.4 kb) carrying *erm* (C). Importantly, the major resistance determinants of the non-NRCS-A clone [*mecA*, *ermA*, *fusB*, *dfrS1 *and *aac (6')-Ie/aph (2'')-Ia*] were chromosomally integrated within SCC*mec* IIIa rather than plasmid-borne, while *blaZ* and aminoglycoside resistance genes were carried on plasmids. The co-carriage of multiple resistance-encoding plasmids, together with the SCC*mec* IIIa chromosomal element, likely underlies the broader antibiotic resistance profile of the non-NRCS-A pulsotype 1300 clone compared with the NRCS-A strains.

This study is among the few evaluating the role of *S. capitis* in LOS in NICU settings in Taiwan. During the study period, we found that the episode number of LOS caused by *S. capitis* in neonates increased slightly year by year in our hospital. Consistent with previous reports [11, 32, 39], most episodes occurred in very low birth weight (<1,500 g) and preterm infants (<32 weeks’’ gestational age). Central venous catheter-associated bacteraemia was again documented in this study.

In the previous study, Felgate *et al*. [21] showed that NRCS-A strains commonly colonized uninfected babies in NICU and may cause subsequent bacteraemia with an indistinguishable strain. In this study, we further correlated molecular genotypes of *S. capitis* with clinical features in NICU infants. Of the 49 isolates, 2 clones were identified, with a non-NRCS-A (85.7%) strain being predominant. Of note, thrombocytopenia was present in 40.8% of episodes, and an elevated CRP level at onset was also noted, even more than one-third of the episodes with a value of >40 mg l^−1^, indicating clinical significance of *S. capitis* LOS. A higher rate of leucocytosis and CRP elevation was observed in infants infected with the NRCS-A strain compared with those infected with non-NRCS-A, though not statistically significant. All 49 isolates were oxacillin resistant, consistent with global findings, and penicillin resistant, with no vancomycin resistance or heteroresistance, similar to those in New Zealand [12]. Despite uniform vancomycin susceptibility, persistent bacteraemia in nine episodes (18.4%) suggested the potential heteroresistance. In addition, the major clone, pulsotype 1300/SCC*mec* III (non-NRCS-A strain), had additional resistance to clindamycin and erythromycin.

There are several limitations in this study. First, this study was conducted in a single medical centre, though the largest hospital in Taiwan; it may still be partly on behalf of the current status of *S. capitis* strains in NICUs in Taiwan. Secondly, infants with only one blood culture positive for *S. capitis* were included, based on a previous study that demonstrated no significant difference between confirming CoNS bacteraemia with one or two positive cultures [40]. However, there remains a possibility of collecting contamination rather than true infection. Nevertheless, all infants exhibited evident clinical symptoms/signs that captured the attention of physicians, leading to the administration of a minimum 5-day course of antimicrobial therapy. Furthermore, in six out of seven episodes, multiple *S. capitis* isolates from a single episode were genetically identical, while the remaining one episode revealed a close subtype. These findings suggest a high likelihood of true infection.

## Conclusions

More than 95% of *S. capitis* bloodstream isolates from neonates in our NICUs, a large medical centre in northern Taiwan, were resistant to oxacillin and penicillin and belonged to two dominant clones, while a very diverse genetic background was identified among those from non-neonates. Among the neonatal isolates, the NRCS-A strain accounted for approximately one-fifth, while a non-NRCS-A strain, with even more resistant genes, dominated for the remaining isolates. No statistically significant differences were found between the two clones in terms of clinical symptoms and laboratory data in neonates.

## Supplementary material

10.1099/mgen.0.001798 Supplementary Material 1.

10.1099/mgen.0.001798 Supplementary Material 2.

## Abbreviations

CARD: Comprehensive Antibiotic Resistance Database
CoNS: coagulase-negative staphylococci
MDR: multidrug resistant
NICUs: neonatal intensive care units
PVL: Panton–Valentine leukocidin
SCC*mec*: staphylococcal chromosome cassette *mec*
SCNs: special care nurseries
TMP-SMX: trimethoprim/sulfamethoxazole
WGS: whole-genome sequencing
