# Supplementary material for: Whole-genome sequencing of Staphylococcus capitis isolates from neonates with healthcare-associated bacteraemia in neonatal intensive care units in Taiwan: comparison between the NRCS-A strain and a non-NRCS-A multi-resistant strain
Source: Microb Genom. 2026 Jul 29;12(7):001798. doi: 10.1099/mgen.0.001798 (PMC13435550; doi:10.1099/mgen.0.001798)
Supplement: Supplementary file 1 [file mgen-12-01798-s001.pdf]

**Supplemental materials**

# Molecular characteristics and clinical features of *Staphylococcus capitis* healthcare-associated late-onset bacteremia among infants hospitalized in neonatal intensive care units: comparison between NRCS-A strain and non NRCS-A strain

Yu-Rou Yeh[a], Yi-Hsuan Huang[b], Chih-Jung Chen[b], Rey-In Lien[b], Ming-Chou Chiang[b], Yhu-Chering Huang[b]

[a]Department of Medical Education, Chang Gung Memorial Hospital at Linkou, Kweishan, Taoyuan, Taiwan

[b]Department of Pediatrics, Chang Gung Memorial Hospital at Linkou, Kweishan, Taoyuan, Taiwan and Chang Gung University School of Medicine, Kweishan, Taoyuan, Taiwan

Supplementary Table 1. Yearly isolates of *Staphylococcus capitis* identified from inpatients hospitalized at Chang Gung Memorial Hospital between 2014 and 2021

| Year | No. isolates | Oxacillin-resistance | | Neonates or not | |
|---|---|---|---|---|---|
| | | ORSC (n=163) | OSSC (n=28) | Neonates* (n=73) | Non-neonates (n=118) |
| 2014 | 19 | 17 | 2 | 6 | 13 |
| 2015 | 19 | 16 | 3 | 7 | 12 |
| 2016 | 21 | 15 | 6 | 7 | 14 |
| 2017 | 23 | 20 | 3 | 8 | 15 |
| 2018 | 25 | 21 | 4 | 9 | 16 |
| 2019 | 23 | 20 | 3 | 11 | 12 |
| 2020 | 30 | 27 | 3 | 11 | 19 |
| 2021 | 31 | 27 | 4 | 14 | 17 |
| Total | 191 | 163 | 28 | 73 | 118 |

ORSC, oxacillin-resistant *S. capitis*; OSSC, oxacillin-sensitive *S. capitis*

*Of the 73 isolates from neonates, 70 isolates (96%) were oxacillin-resistant.

Supplementary Table 2. Comparison of antibiotic susceptibility of 191 *Staphylococcus capitis* isolates, stratified by neonates or not

| Antibiotics | No. neonates (%) (n=73) | No. non-neonates (%) (n=118) | P value |
|---|---|---|---|
| Clindamycin | 16 (21.9) | 48 (40.7) | 0.008* |
| Erythromycin | 14 (19.2) | 49 (41.5) | 0.001* |
| Oxacillin | 3 (4.1) | 25 (21.2) | 0.001* |
| Penicillin | 0 (0) | 5 (4.2) | 0.158 |
| SXT | 67 (91.8) | 102 (86.4) | 0.261 |
| Teicoplanin | 73 (100) | 115 (97.5) | 0.288 |
| Vancomycin | 73 (100) | 118 (100) | 1 |
| Rifampin | 73 (100) | 92 (78) | <0.001* |

SXT, trimethoprim/sulfamethoxazole

Data are presented as n (%). Pearson's chi-square test or Fisher's exact test was used as appropriate.

*$p<0.05$

Supplementary Table 3. Distribution of yearly isolates of 73 *S. capitis* blood isolates from infants hospitalized at neonatal units in CGMH between 2014 and 2021, stratified by pulsotypes

| Year | Pulsotypes, No. of isolates | | | | |
|---|---|---|---|---|---|
| | Total | 100 | 300 | 1300 | 2900 |
| 2014 | 6 | 0 | 0 | 6 | 0 |
| 2015 | 7 | 0 | 0 | 7 | 0 |
| 2016 | 7 | 0 | 0 | 6 | 1 |
| 2017 | 8 | 1 | 1 | 6 | 0 |
| 2018 | 9 | 0 | 4 | 5 | 0 |
| 2019 | 11 | 0 | 4 | 7 | 0 |
| 2020 | 12 | 0 | 5 | 6 | 0 |
| 2021 | 13 | 0 | 2 | 11 | 0 |
| Total | 73 | 1 | 16 | 55 | 1 |

Supplementary Table 4. Virulence genes detected in 12 blood isolates of *Staphylococcus capitis* strains

| Strain | clade | *sdrC* | *sdrD* | *sdrE* | sdrH |
|---|---|---|---|---|---|
| SC20 | D | 98.75 | 90.8 | | |
| SC108 | A | | | | 92.09 |
| SC116 | B | | | 90.35 | |
| SC117 | D | 90.64 | | 90.18 | |
| SC121 | C | 90.71 | | | |
| SC127 | C | | | 91.24 | |
| SC130 | C | 97.5 | 90.27 | | |
| SC131 | C | 90.72 | | | |
| SC132 | C | 90.7 | | | |
| SC149 | A | | | 90.06 | |
| SC151 | ND | 90.15 | | | |
| SC158 | B | | | | 90.54 |

The empty cells indicate no detection of the specified virulence gene. The numbers in the cells represent the percentages of identity between detected sequences and the reference sequence of the specified gene in the VFDB database.

ND signifies not determined. The strain does not belong to any of the four major clades.

Supplementary Table 5. Comparison of antibiotic susceptibility of 49 neonatal *Staphylococcus capitis* isolates, stratified by pulsed-field gel electrophoresis types (pulsotypes)

| Antibiotics | Pulsotype, No. (%) of subjects | | | p-value |
|---|---|---|---|---|
| | Total (n=49) | 1300 (n=42) | 300 (n=7) | |
| Clindamycin | 7 (14.3) | 0 (0) | 7 (100) | 0.000* |
| Erythromycin | 6 (12.2) | 0 (0) | 6 (85.7) | 0.000* |
| Oxacillin | 0 (0) | 0 (0) | 0 (0) | 1 |
| Penicillin | 0 (0) | 0 (0) | 0 (0) | 1 |
| SXT | 42 (85.7) | 35 (83.3) | 7 (100) | 0.573 |
| Teicoplanin | 49 (100) | 42 (100) | 7 (100) | 1 |
| Vancomycin | 49 (100) | 42 (100) | 7 (100) | 1 |
| Linezolid | 49 (100) | 42 (100) | 7 (100) | 1 |
| Rifampin | 49 (100) | 42 (100) | 7 (100) | 1 |

SXT, trimethoprim/sulfamethoxazole

Data are presented as n (%). Pearson's chi-square test or Fisher's exact test was used as appropriate.

*$p$<0.05

Supplementary Table 6. Empirical antibiotic used for 49 episodes of late-onset sepsis due to Staphylococcus capitis, stratified by PFGE types

| Empirical antibiotic treatment | No. (%) of subjects | | | p-value |
|---|---|---|---|---|
| | Total (n=49) | 1300 (n=42) | 300 (n=7) | |
| Combination therapy | | | | |
| b-lactam + aminoglycoside | 5 (10.2) | 5 (11.9) | 0 (0) | 1.000 |
| b-lactam + third-generation cephalosporin | 5 (10.2) | 4 (9.5) | 1 (14.3) | 0.554 |
| b-lactam + glycopeptide | 3 (6.1) | 3 (7.1) | 0 (0) | 1.000 |
| b-lactam + aminoglycoside | 1 (2.0) | 1 (2.4) | 0 (0) | 1.000 |
| b-lactam + carbapenem | 1 (2.0) | 0 (0) | 1 (14.3) | 0.143 |
| Glycopeptide + aminoglycoside | 2 (4.1) | 2 (4.8) | 0 (0) | 1.000 |
| Glycopeptide + third-generation cephalosporin | 14 (28.6) | 12 (28.5) | 2 (28.6) | 1.000 |
| Glycopeptide + carbapenem | 1 (2.0) | 0 (0) | 1 (14.3) | 0.143 |
| Third-generation cephalosporin + first-generation cephalosporin | 1 (2.0) | 1 (2.4) | 0 (0) | 1.000 |
| Third-generation cephalosporin + aminoglycoside | 1 (2.0) | 1 (2.4) | 0 (0) | 1.000 |
| Metronidazole + aminoglycoside | 1 (2.0) | 0 (0) | 1 (14.3) | 0.143 |
| Metronidazole + glycopeptide + first-generation cephalosporin | 1 (2.0) | 1 (2.4) | 0 (0) | 1.000 |
| Glycopeptide + third-generation cephalosporin + b-lactam + carbapenem | 1 (2.0) | 1 (2.4) | 0 (0) | 1.000 |
| Glycopeptide + third-generation cephalosporin + metronidazole | 1 (2.0) | 1 (2.4) | 0 (0) | 1.000 |
| Monotherapy | | | | |
| Glycopeptide | 9 (18.4) | 8 (19.0) | 1 (14.3) | 1.000 |
| Aminoglycoside | 1 (2.0) | 1 (2.4) | 0 (0) | 1.000 |
| Carbapenem | 1 (2.0) | 1 (2.4) | 0 (0) | 1.000 |

b-lactam included oxacillin, ampicillin; aminoglycosides included gentamicin and amikacin; third-generation cephalosporins included cefotaxime, and ceftazidime; glycopeptides included vancomycin and teicoplanin; carbapenems included meropenem and ertapenem.

Supplementary Table 7. Definite antibiotic used in 49 episodes of late-onset sepsis due to Staphylococcus capitis, stratified by PFGE types.

| Definite antibiotic treatment | No. (%) of subjects | | | p-value |
|---|---|---|---|---|
| | Total (n=49) | 1300 (n=42) | 300 (n=7) | |
| Combination therapy | | | | |
| Vancomycin + aminoglycoside | 4 (8.2) | 4 (9.5) | 0 (0) | 1.000 |
| Vancomycin + third-generation cephalosporin | 4 (8.2) | 4 (9.5) | 0 (0) | 1.000 |
| Vancomycin + metronidazole + third-generation cephalosporin | 1 (2.0) | 1 (2.4) | 0 (0) | 1.000 |
| Vancomycin + carbapenem | 5 (10.2) | 3 (7.1) | 2 (28.6) | 0.143 |
| Vancomycin + metronidazole | 1 (2.0) | 1 (0) | 0 (0) | 1.000 |
| Vancomycin + SXT | 1 (2.0) | 0 (0) | 1 (14.3) | 0.143 |
| Monotherapy | | | | |
| Vancomycin | 30 (61.2) | 26 (61.9) | 4 (57.1) | 1.000 |
| Outcome | | | | |
| Persistent | 9 (18.4) | 7 (16.7) | 2 (28.6) | 0.598 |
| Recovery | 39 (79.6) | 34 (81.0) | 5 (71.4) | 0.620 |

SXT, trimethoprim/sulfamethoxazole

Aminoglycosides included gentamicin and amikacin; third-generation cephalosporins included cefotaxime, and ceftazidime; carbapenems included meropenem and ertapenem.
